# Minimally Invasive Neurosurgery for Spontaneous Intracerebral Hemorrhage—10 Years of Working Progress at National Taiwan University Hospital

**DOI:** 10.3389/fneur.2022.817386

**Published:** 2022-05-20

**Authors:** Chiu-Hao Hsu, Sheng-Chieh Chou, Lu-Ting Kuo, Sheng-Jean Huang, Shih-Hung Yang, Dar-Ming Lai, Abel Po-Hao Huang

**Affiliations:** ^1^Department of Surgery, National Taiwan University Hospital Hsin-Chu Branch, Biomedical Park Hospital, Hsin-Chu, Taiwan; ^2^Graduate Institute of Clinical Medicine, College of Medicine, National Taiwan University, Taipei, Taiwan; ^3^Division of Neurosurgery, Department of Surgery, National Taiwan University Hospital, and National Taiwan University College of Medicine, Taipei, Taiwan; ^4^Division of Hematology, Department of Internal Medicine, National Taiwan University Hospital, and National Taiwan University College of Medicine, Taipei, Taiwan

**Keywords:** functional outcome, intracerebral hemorrhage, minimally invasive neurosurgery, mortality, early surgery

## Abstract

Intracerebral hemorrhage (ICH) is a life-threatening disease with a global health burden. Traditional craniotomy has neither improved functional outcomes nor reduced mortality. Minimally invasive neurosurgery (MIN) holds promise for reducing mortality and improving functional outcomes. To evaluate the feasibility of MIN for ICH, a retrospective analysis of patients with ICH undergoing endoscopic-assisted evacuation was performed. From 2012 to 2018, a total of 391 patients who underwent ICH evacuation and 76 patients who received early (<8 h) MIN were included. The rebleeding, mortality, and morbidity rates were 3.9, 7.9, and 3.9%, respectively, 1 month after surgery. At 6 months, the median [interquartile range (IQR)] Glasgow Coma Scale score was 12 (4.75) [preoperative: 10 (4)], the median (IQR) Extended Glasgow Outcome Scale score was 3 (1), and the median (IQR) Modified Rankin Scale score was 4 (1). The results suggested that early (<8 h) endoscope-assisted ICH evacuation is safe and effective for selected patients with ICH. The rebleeding, morbidity, and mortality rates of MIN in this study are lower than those of traditional craniotomy reported in previous studies. However, the management of intraoperative bleeding and hard clots is critical for performing endoscopic evacuation. With this retrospective analysis of MIN cases, we hope to promote the specialization of ICH surgery in the field of MIN.

## Introduction

The optimal treatment for intracerebral hemorrhage (ICH) remains among the most controversial topics in neurosurgery. ICH is associated with high morbidity and mortality rates and imposes a substantial economic burden worldwide. Recent studies have reported that compared with traditional craniotomies, minimally invasive treatments resulted in more favorable outcomes (1–3). These treatments include minimally invasive clot evacuation with stereotactic or endoscopic aspiration with or without thrombolytic usage. Minimally invasive treatments cannot only reduce mortality but also improve neurological recovery in selected patients (3–5), which is rarely observed and encouraging for the treatment of ICH.

Taiwan has a highly dense population and many hospitals. Patients with ICH can arrive at the hospital shortly after ictus. This is especially true in Taipei City, the capital of Taiwan, where our hospital, National Taiwan University Hospital (NTUH), is located. The majority of surgeries were performed within 4 h after ictus (6). We have performed minimally invasive neurosurgery (MIN) under endoscopic guidance for ICH evacuation since 2008. For more than 10 years, we have been continuously refining the surgical technique, equipment, protocol, and workflow in the pursuit of clinical excellence and improved outcomes. With the paradigm shifting toward minimally invasive surgery, we share our experience in this retrospective analysis.

## Materials and Methods

### Study Design

This retrospective study was conducted at NTUH after obtaining the approval from the Ethical Review Board of our institute. We extracted and analyzed clinical data from the medical charts of patients with spontaneous supratentorial ICH who received early MIN (within 48 h after ictus) from 2012 to 2018. This study was performed in compliance with applicable local regulations and the ethical principles of the Declaration of Helsinki. All the experimental protocols were approved by the Institutional Review Board of NTUH. Because of the retrospective nature of this study, the requirement of informed consent was waived. Informed consent for surgery was obtained from each patient per routine practice.

### Patient Selection

A series of experiences presented in this study represents the working progress of 6 years following a previously published series of our experiences from 2008 to 2011 (6). Between 2012 and 2018, a total of 391 patients underwent ICH evacuation at NTUH. We included patients with spontaneous supratentorial ICH who received early MIN (within 48 h after ictus). A cutoff of <48 h was selected on the basis of the finding of a meta-analysis that patients who underwent MIN within 72 h were two times more likely to achieve functional independence (7). In this experience, ICH evacuation after 48 h of ictus may lead to significant brain edema and massive blood clots that favor decompressive craniectomy as the primary treatment instead of MIN.

We excluded patients with etiology related to structural lesions or nonprimary lesions (cerebral aneurysm, arteriovenous malformation, and cavernous malformation), cerebellar hemorrhage, traumatic ICH, tumor bleeding, hemorrhagic transformation after ischemic stroke or postoperative bleeding, or primary intraventricular hemorrhage (IVH). In addition, we excluded patients who underwent traditional craniotomy, external ventricular drainage, or decompressive craniectomy as the primary treatment.

**Figure 1:**
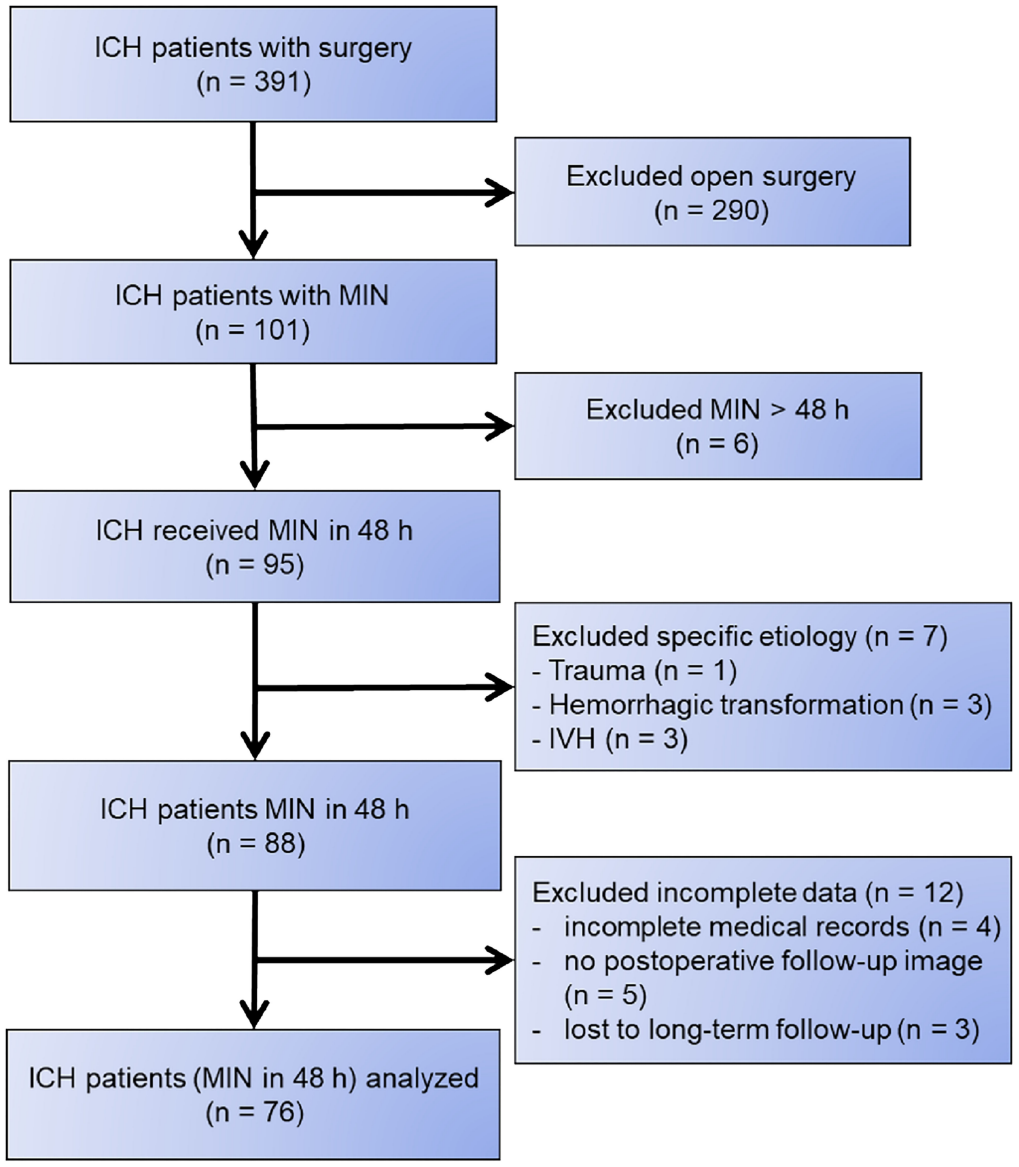
Flowchart of patient selection.

We carefully reviewed the medical records of the remaining 88 patients who underwent minimally invasive endoscope-assisted ICH evacuation. Furthermore, we excluded 12 patients because they had incomplete medical records (*n* = 4), did not undergo postoperative follow-up imaging (*n* = 5), or were lost to long-term follow-up (*n* = 3). Finally, 76 patients were included in this study (Figure 1).

### Rationale for Surgical Management of Patients With Intracerebral Hemorrhage

Currently, we have four attending neurosurgeons in the NTUH ICH team, which is the only dedicated ICH team in the country. This specialized team aims to improve functional outcomes, quality of care, and quality of life for patients. The minimally invasive endoscopic-assisted ICH evacuation was performed in approximately 70% of our surgical cases. On the basis of our extensive experience and the literature review, we have developed guidelines for the optimal treatment of patients with ICH in NTUH. Recent studies have suggested that early surgery for ICH is associated with satisfactory functional outcomes and low mortality (8–12). This finding is concordant with our practice at NTUH where we performed surgery in more than 80% of patients with ICH within 4 h after ictus (6). We prefer the endoscopic-assisted evacuation over stereotactic thrombolytic therapy because of its earlier decompression, high hematoma clearance rate, and cost-effectiveness (13).

The selection of the surgical method depended on our clinical experience and the literature review. MIN was mostly performed for deep-seated early ICH (such as the basal ganglia and thalamus, with ICH volume ranging from 20 to 80 ml). For superficial (subcortical or lobar) ICH, minicraniotomy using microscopic evacuation or endoscopic-assisted MIN was both feasible. For delayed cases (>72 h after ictus), craniectomy with ICH evacuation was preferred because such cases usually have substantial brain edema and massive blood clots that favor decompressive craniectomy as the primary treatment instead of MIN.

### Surgical Procedure

Various MIN techniques have been developed for ICH evacuation (14). We adopted the endoscopic-assisted approach where suction is applied on the side of the endoscope inside a 10–12-mm port or sheath that creates the working space (also known as the port-based approach). By contrast, the pure endoscopic approach [e.g., the stereotactic intracerebral hemorrhage underwater blood aspiration (SCUBA) technique] uses the aspiration device inside the endoscope (through its working channel) with a 19-F peel-away sheath.

Clot evacuation could be smoothly performed with one hand holding the endoscope and the other hand holding a suction device. We used 8-Fr suction to remove the clot because suction with a smaller caliber would lead to the ineffective removal of ICH. In the presence of a hard clot, the alligator punch was used to pass through the sheath for piecemeal removal. In extreme cases, we used the cavitron ultrasonic surgical aspirator (CUSA) to remove the hard clot. The CUSA is a surgical device used in neurosurgical procedures. The CUSA is coupled with low-frequency ultrasound that helps operators to dissect or fragment tissues or pathologies. The CUSA was used to manage ICH cases with hard clots that could not be removed with suction only. Apollo, Artemis, and Myriad are not available in Taiwan; thus, we have no experience in using these devices.

Regarding hemostasis during surgery, we initially used an endoscopic bipolar or a suction coagulation device; however, these techniques were time-consuming. Moreover, achieving hemostasis by using these techniques was difficult. Later, we used local hemostatic agents that remarkably reduced the time of hemostasis, with a low rebleeding rate. In our experience, no active bleeding requiring coagulation was noted in more than 90% of cases and local hemostatic agents [e.g., FLOSEAL Hemostatic Matrix (Baxter Healthcare Corporation, Fremont, California, USA), a gelatin–thrombin matrix] yielded satisfactory hemostasis (15).

When an active bleeder was encountered during surgery, we suctioned out blood, used irrigation to identify the active bleeder, contacted it with the suction tip, and performed cauterization by using a metallic suction tube. The importance of a balanced suction irrigation technique in the minimally invasive surgery of ICH was described in detail in a previous study (16). After hemostasis, we placed an external ventricular drain if the frontal horn was entered during surgery; otherwise, we inserted an intracranial pressure (ICP) monitor [fiber optic devices (e.g., Camino ICP Monitor) and strain gauge devices (e.g., Codman Microsensor and Raumedic Neurovent-P ICP sensor)] through the surgical corridor created by the trocar under direct vision in the perihematoma zone of the brain parenchyma. A surgical biopsy of the brain parenchyma was performed, if amyloid angiopathy was suspected (17).

In terms of surgical techniques, we recommend using the penetrating technique (Huang's technique) instead of the circumferential technique used in our previous studies (6, 8) for ICH removal because it prevents collateral brain damage when using the trocar or port inside the normal brain tissue. For typical basal ganglia ICH, we used modified Kocher's point (1 cm lateral to Kocher's point). A 2.5-cm incision was vertically made on modified Kocher's point, creating a 1.5–2.0-cm burr hole. After dural opening, either the trans-sulcal or transcortical approach was used. The intraoperative Aloka burr-hole type ultrasound was selectively used to verify the trajectory and depth of ICH before puncturing with the trocar (we used a transparent trocar with an outer diameter of 12 mm and length of 10 cm for putaminal ICH) (6). For thalamic ICH, we used a considerably different surgical approach. Initially, our team used an aggressive approach involving the use of the endoscopic technique to remove thalamic ICH in the brain parenchyma. However, the surgical outcome was poor and most of the patients remained comatose after evacuation. Therefore, our current goal was to relieve hydrocephalus and remove IVH to minimize shunt dependency. We only dived into thalamic ICH in the brain parenchyma when we observed the rupture side during endoscopic surgery. The first step was to determine whether to use the frontal or occipital approach; we occasionally used the contralateral approach (18). The rupture side could be determined through preoperative CT performed to choose the surgical approach. The thalamostriate vein was required to be carefully preserved to prevent iatrogenic infarction.

We applied the concepts of the SCUBA technique in our MIN for ICH evacuation. The SCUBA technique differs from minimally invasive ICH intervention because it combines two separate neuroendoscopic strategies in two phases: the first is using dry-field conditions and the second is using a wet-field strategy (19). This prevents the collapse of the hematoma cavity and allows for complete clot removal. This technique is useful for bleeder identification. During the procedure, balancing suction and irrigation is crucial for bleeder identification and hemostasis (16).

For brain access, most neurosurgeons used the 12-mm outer diameter transparent trocar. The peel-away sheath was occasionally used by neurosurgeons who were comfortable with ventriculoscopic techniques (6). In our experience, only 10%−15% of the cases had intraoperative bleeding requiring hemostasis and most of the small arterial bleeding from thalamostriate perforators could be stopped using local hemostatic agents (8). During surgery, similar to the SCUBA technique, we alternated between air and water phases; we found this technique to be useful for preventing the hematoma cavity from collapsing with resultant residual ICH and for identifying the bleeder (19). Cauterization was required in <5% of cases and was easily performed using traditional suction at the bleeding point (vessel) with unipolar cauterization touching the handle of the sucker (poor man's suction bipolar). Alternatively, commercialized suction bipolar devices can be used (6). If all these attempts failed, the next step was to change to a larger trocar (Vycor, with a diameter of >2 cm used for traditional bipolar cauterization). We rarely converted to craniotomy, which was only performed in three out of more than 400 ICH cases.

### Perioperative Care

In the perioperative period, we observed that dexmedetomidine substantially reduced blood pressure fluctuations. This reduction is associated with a decreased rebleeding rate, as demonstrated in various experiences and other major surgeries (20). The effect of dexmedetomidine on patients with ICH was reported in a clinical trial (21). For cases with a high risk of rebleeding, we may consider tranexamic acid and recombinant factor VIIa (NovoSeven) (22). Currently, we routinely perform CT within 24 h after surgery to confirm the residual hematoma and the degree of evacuation.

Cognitive enhancers and neural stimulants were reported to improve cognitive and behavioral impairments in patients with putaminal ICH and traumatic brain injury (23). We used methylphenidate in patients with ICH who were comatose before the surgery to improve neurological outcomes. In our experience of 58 patients, methylphenidate was safe and effective and was associated with faster consciousness recovery, greater chance of successful extubation, and shorter intensive care unit (ICU) stay. However, a study reported the rare side effects of methylphenidate, such as serotonin syndrome, in patients with ICH (24).

### Data Collection

We collected data on the following characteristics of the patients: hematoma location and volume, presence of IVH, sex, age, time of operation, operative blood loss, and hematoma evacuation rate. All the patients underwent preoperative head CT and a follow-up head CT within 1 week after surgery. The estimated hematoma volume was calculated using the ABC/2 method (A: maximum length in the axial cut of CT, B: width perpendicular to A on the same CT cut, and C: the number of slices multiplied by the slice thickness).

### Clinical Outcome

Outcome measures included the hematoma evacuation rate, rebleeding, mortality rate, morbidity rate, the preoperative and postoperative Glasgow Coma Scale (GCS) scores, the postoperative Extended Glasgow Outcome Scale (GOSE) scores, the Modified Rankin Scale (mRS), length of ICU stay, and the length of hospital stay. The hematoma evacuation rate was calculated as [(preoperative volume – postoperative volume)/(preoperative volume) × 100 (%)]. Mortality was defined as all-cause death occurring within 30 days after surgery. Rebleeding and morbidity rates were examined 1 month after surgery. Rebleeding was defined as a postoperative hematoma volume greater than the preoperative volume or a difference of <5 ml between preoperative and postoperative hematoma volume. Morbidity included wound dehiscence and surgical site infection, including meningitis, ventriculitis, and brain abscess. Postoperative outcomes included the GCS scores at 1 and 6 months and the GOSE and the mRS scores at 6 months.

### Statistical Analysis

Descriptive statistics were used to present categorical data in numbers and percentages and continuous data in numbers, medians, and interquartile ranges (IQRs). The surgical and functional outcomes were analyzed using the Kruskal–Wallis test. A *P*-value of <0.05 was regarded as statistically significant. Statistical analyses were performed using SPSS (version 25, Chicago, Illinois, USA).

## Results

### Demographics and Baseline Characteristics

Table 1 summarizes the demographics of the patients. We divided the patients into three groups according to the hematoma location: putaminal (*n* = 56), thalamic (*n* = 9), and subcortical (*n* = 11). The male predominance of approximately 69.7% was noted and IVH was found in 47.4% of the patients. Most of the enrolled patients were aged from 57 to 60 years, except for those with thalamic ICH (median age: 66 years). The median operative time ranged from 104 to 108 min. The ICH score (median: 2) and operative blood loss (median: 50 ml) were similar among the different types of ICH. The patients with putaminal ICH had the highest preoperative ICH volume (median: 45 ml).

**Table 1:** Characteristics of patients.

	**Putaminal**	**Thalamic**	**Subcortical**	**All**
	**(*n* = 56)**	**(*n* = 9)**	**(*n* = 11)**	
Male, *n* (%)	42 (75.0)	5 (55.6)	6 (54.5)	53 (69.7)
Age (year), median (IQRs)	58 (17.25)	66 (6)	62 (32)	59 (18)
ICH score, median (IQRs)	2 (1)	3 (1)	2 (1.5)	2 (1)
IVH, *n* (%)	26 (46.4)	6 (66.7)	4 (36.4)	36 (47.4)
**Anticoagulants and antiplatelets**, ***n*** **(%)**				
Antiplatelet agents	6 (10.7)	1 (11.1)	3 (27.3)	10 (13.2)
Anticoagulant agents	1 (1.8)	0 (0)	0 (0)	1 (1.3)
Both	2 (3.6)	0 (0)	0 (0)	2 (2.6)
Operative time (min), median (IQRs)	108 (50.25)	109 (63)	104 (34.5)	107 (50.25)
Preoperative ICH volume (ml), median (IQRs)	45 (28.75)	35 (15)	50 (20)	42.5 (25)
Operative blood loss (ml), median (IQRs)	50 (12.5)	50 (0)	50 (0)	50 (0)
ICU length of stay (day), median (IQRs)	16 (14.5)	13.5 (15.25)	19 (3)	16 (13)
Hospital length of stay (day), median (IQRs)	30 (23.5)	29 (25.25)	37 (21)	28 (11.5)

*IVH, intraventricular hemorrhage; IQR, interquartile range; ICU, intensive care unit; ICH, intracerebral hemorrhage*.

Most of the patients (97%) underwent MIN within 4 h after ictus. Only two patients underwent surgery on the second day of ictus because of hematoma expansion. Our hospital defines three classes for emergent operation. The duration between notifying the operation room to the start of operation is limited to 30 min, 2 h, and 4 h for first-class, second-class, and third-class emergent operations, respectively. ICH is classified as a third-class surgery, which is mostly performed within 4 h.

### Postoperative Outcomes

Table 2 summarizes the surgical and functional outcomes, with no significant difference noted between the groups. The median (IQR) hematoma evacuation rate was 85.7% (16.7%) and the median (IQR) postoperative ICH volume was 5 (5) ml. According to the Minimally Invasive Surgery Plus Recombinant Tissue Plasminogen Activator for Intracerebral Hemorrhage Evacuation III (MISTIE III) study, a reduction in clot size to ≤ 15 ml is associated with improvement in functional outcomes (4). In our series, 64 (84.2%) patients reached the goal of a residual hematoma volume of <15 ml. The overall rebleeding rate at 1 month after surgery was 3.9%. Three patients experienced rebleeding in the first postoperative follow-up [2 (3.6%) in the putaminal group and 1 (11.1%) in the thalamic group]. Six patients died eventually (the overall mortality rate: 7.9%): two patients died from pneumonia and sepsis, two patients died from postoperative central nervous system infection, and two patients died because their family members decided to withdraw life support owing to the lack of clinical improvement. The morbidity rate at 1 month after surgery was 3.9%; one patient developed scalp wound dehiscence, one patient developed meningitis and ventriculitis, and one patient developed brain abscess.

**Table 2:** Outcomes.

	**Putaminal**	**Thalamic**	**Subcortical**	***P*-Value**	**All**
	**(*n* = 56)**	**(*n* = 9)**	**(*n* = 11)**		**(*n* = 76)**
Rebleeding, *n* (%)	2 (3.6)	1 (11.1)	0 (0)		3 (3.9)
Mortality, *n* (%)	5 (8.9)	0 (0)	1 (9.1)		6 (7.9)
Morbidity, *n* (%)	3 (5.4)	0 (0)	0 (0)		3 (3.9)
Hematoma evacuation rate (%), median (IQRs)	86.6 (11.9)	75.0 (19.0)	80.0 (20.8)	0.170	85.7 (16.7)
Postoperative ICH volume (ml), median (IQRs)	5 (5)	10 (5)	10 (12.5)		5 (5)
Postoperative hematoma volume <15 ml, *n* (%)	48 (85.7)	8 (88.8)	8 (77.8)		64 (84.2)
**GCS, median (IQRs)**					
Pre-op	10 (4)	8 (3)	11 (3.5)	0.343	10 (4)
Post-op 1 month	11 (4)	12 (5)	13 (4)	0.508	12 (4)
Post-op 6 months	12 (4)	14 (6)	14 (3)	0.659	12 (4.75)
**GOSE, median (IQRs)**					
Post-op 6 months	3 (1)	4 (2)	4 (1.5)	0.666	3 (1)
**mRS, median (IQRs)**					
Post-op 6 months	4 (1)	4 (2)	4 (1)	0.997	4 (1)
mRS 0–2, *n* (%)	7 (12.5)	1 (11.1)	3 (22.2)		11 (14.5)
mRS 0–3, *n* (%)	21 (37.5)	4 (44.4)	3 (22.2)		28 (36.8)

*GCS, Glasgow coma scale; GOSE, Glasgow outcome scale extended; ICU, intensive care unit; mRS, modified rankin scale; OP, operation; IQR, interquartile range; ICH, intracerebral hemorrhage*.

Functional outcomes improved after surgery, with no significant differences observed between the groups (Table 2). The median (IQR) preoperative GCS score was 10 (4), which was numerically increased to 12 (4) at 1 month and 12 (4.75) at 6 months. The median (IQR) GOSE score at the 6-month follow-up was 3 (1). The median (IQR) mRS score at the 6-month follow-up was 4 (1). Satisfactory outcomes (mRS score: 0–3) were noted in 36.8% of the patients.

## Discussion

The prevalence of ICH is especially high in Asia-Pacific regions, where ICH accounts for approximately 30%−40% of all stroke cases. In the United States, ICH accounts for approximately only 15% of all stroke cases (25). Each year in China alone, more than 150,000 patients receive minimally invasive treatment for ICH (8). Race and ethnicity appear to explain some of the variation in clinical characteristics and outcomes after acute ICH; for example, Caucasian patients with ICH are more likely to be older, have a larger ICH volume, and have a higher mortality rate than Asian patients (26).

The operation rate considerably varies worldwide, ranging from 2 to 74% (27). In the United States, the early operation rate was previously 16% and decreased to 6% in 2005, presumably due to the results of the Surgical Treatment for Ischemic Heart Failure (STICH) trial (28, 29). With the current increasing interest in surgical treatment, the operation rate has increased to approximately 20% (1, 30). In the Asia-Pacific region, the operation rate ranges from 30 to 40% and the MIN method, either stereotactic aspiration or endoscopic-assisted evacuation, is widely used (27).

### Clinical Outcomes Compared With Prior Minimally Invasive Neurosurgery Reports

In our series, early MIN within 48 h after ICH ictus resulted in a low rebleeding rate (3.9%), low mortality rate (7.9%), and low morbidity rate (3.9%); moreover, 36.8% of the patients exhibited satisfactory functional outcomes (mRS score: 0–3) 6 months after MIN. Although the overall median GOSE score indicated that the patients still required assistance to perform the activities of daily living (GOSE = 3) 6 months after MIN, the patients with thalamic or subcortical ICH could occasionally be at home independently (GOSE = 4) and their overall GCS score improved over time. Compared with those of patients included in other MIN studies, the postoperative outcomes of our patients appeared to be more favorable. In the MISTIE III study (4), 44% of patients achieved the mRS score of 0–3, 39% of patients had the GOSE score of ≥4 at 12 months, and the mortality rate at 6 months was 15%. In the MISTIE III study, 33.4% of patients achieved the mRS score of 0–3 and the mortality rate within 30 days was 14.8% (31). In the intraoperative stereotactic CT-guided endoscopic surgery (ICES) trial including six patients, 24% of patients achieved the mRS score of 0–3 and no patient died within 30 days after surgery (3).

### Literature Support for Minimally Invasive Neurosurgery Over Traditional Craniotomy for Intracerebral Hemorrhage Evacuation

In patients with supratentorial ICH, compared with medical management alone, surgery in addition to medical management reduced functional dependency and mortality more effectively (1, 32). Moreover, two meta-analyses have demonstrated that selected patients benefited from MIN over other treatments (7, 33). The first meta-analysis of five randomized trials and nine prospective studies observed a significant difference in the mortality rate between patients receiving MIN and those receiving traditional craniotomy [odds ratio (OR), 0.76, 95% CI, 0.60–0.97] as well as a lower rate of rebleeding and a higher rate of satisfactory neurological recovery for the MIN approach (33). The second meta-analysis of 15 randomized controlled trials reported that patients with ICH who received MIN within 24 h of ictus were 2.8 times more likely to achieve functional independence, whereas patients who received MIN within 72 h were two times more likely to reach functional independence (7).

Traditional craniotomy requires brain retraction and is highly invasive and traumatic. The problem with retraction in neurosurgery should be emphasized and is often avoided, especially in deep-seated ICH, such as putaminal ICH (28, 34). In our experience, retraction may lead to rebleeding. The rebleeding rate in open craniotomy using retraction was 15%−40% (35) and was significantly higher than that of 0%−3.3% in the MIN group (6, 8, 12, 16, 36). Moreover, long-term brain atrophy after brain retraction is alarming because retraction is associated with the chronic local thinning of the neocortex (37).

### Early Minimally Invasive Neurosurgery for Clot Evacuation, Hemostasis, and Low Rebleeding Rate

Rebleeding is the primary concern in MIN and usually occurs within 4 h of ictus. The American Heart Association/American Stroke Association Guidelines for the Management of Spontaneous Intracerebral Hemorrhage in 2015 indicated that ultra-early craniotomy (within 4 h of ictus) was associated with an increased risk of rebleeding. These data were from a study that performed ultra-early craniotomy in 24 patients and the rebleeding rate was 40% (35). Because of the concern of a high rebleeding rate of ultra-early craniotomy, randomized prospective trials have reported a timeframe for surgery that ranges from 6 to 24 h postictus. By contrast, early endoscopic ICH evacuation has been performed in many patients, with low rebleeding rates of 0%−3.3% (6, 8, 12, 16, 36), suggesting that MIN reduces the high rebleeding rate. In our previous study, 84% of cases were operated within 4 h after ictus, resulting in a rebleeding rate of 1.5% (6). In this study, we included the patients using antiplatelet and anticoagulative agents and observed a rebleeding rate of 3.9%. Similarly, Chen et al. (12) treated seven patients within 5 h after ICH and observed no postoperative rebleeding. Nakagasaka treated 23 patients at a median time of 4 h and noted no postoperative rebleeding (16). Nishihara treated 82 patients within 3 h after ICH and observed no postoperative rebleeding (11). Miki et al. treated 127 patients with early (<8 h) endoscopic-assisted ICH evacuation and reported a postoperative rebleeding rate of 7.1% (38).

A meta-analysis of eight studies including 2,186 patients demonstrated the improved functional outcome of surgery performed within 8 h of ictus (10). These findings indicated that early ICH evacuation using MIN may reduce the rebleeding rate in ICH and improve surgical outcomes (9). This finding is in contrast to the notion that early craniotomy for ICH leads to a high rebleeding rate (9, 35). These facts may cause a paradigm shift for neurosurgeons to perform early MIN decompression to reduce secondary brain injuries associated with ICH (e.g., perihematomal edema).

Many studies have enrolled patients with stable clots shown on the CT scan performed 6 h post-onset, including the MISTIE III study, the International Verapamil-Trandolapril Study (INVEST), and the ICES trial (3, 9). The main reason for setting the selection criteria is because a previous study reported that ultra-early surgery within 4 h after ictus was associated with high rebleeding rates (35). However, we must be aware that the majority (25%) of patients with ICH deteriorate within the first few hours of ictus and may require clot evacuation within 4 h after ictus, especially for patients with a spot sign or black hole sign (39).

All the studies have reported that MIN performed within 8 h after ictus reduced the rebleeding rate (Supplementary Table 1). Therefore, we suggest that minimally invasive surgical trials for ICH should not exclude patients operated within 4–6 h; instead, these are the patients with the most favorable functional outcome per our experience of more than 400 patients with MIN. Similarly, a single-arm surgical evaluation in 39 patients with ICH indicated that 52% of the patients achieved functional independence at follow-up and no mortality was noted (40). If surgical evacuation is performed early, secondary injury from ICH may be avoided. Moreover, if early clot evacuation can reduce the rebleeding rate and mass effect, MIN will most likely benefit patients when it is performed early. In short, we strongly recommend early MIN (8 h postictus) for ICH because patients will likely to be benefited from such surgery, especially in terms of functional outcomes.

The ideal approach to surgically treat ICH is to maintain a balance among minimal invasiveness, completeness of clot removal, and secure hemostasis. In our experience, the vital questions are: (1) whether ICH volume can be reduced to <15 ml, which may lead to improved functional outcomes (4); (2) whether intraoperative bleeding is manageable (if traditional cauterization is necessary, if the working space is adequate for hemostasis, and if the trocar or probe needs to be changed to a larger one); and (3) whether progressive brain edema occurs after ICH removal.

### Limitations

This retrospective study has several limitations. First, this single-center study analyzed the data of 76 patients only. These study results lack generalizability and can be applied only to a narrow population or in a very specific situation; therefore, we suggest that MIN should be considered in early deep-seated ICH with volume ranging from 30 to 80 ml. On the basis of the findings of previous studies, MIN should be considered for deteriorating patients with ICH with a clot volume of > 25 ml (33, 41). In our experience, a clot volume of > 80 ml is more difficult to treat minimally invasively. Second, this study did not include the control group to detect the difference between MIN and traditional craniectomy or medical treatment. Third, the patient cohort with a small sample size was heterogeneous with different clinical decision-making among the four different surgeons. Finally, the lack of data integrity is inherent due to the nature of the retrospective study. We did not manage missing data because it reflected a real-world setting. Because of these limitations, these study outcomes should be interpreted with caution. These promising preliminary results warrant further large-scale and well-controlled investigations.

## Conclusion

Early endoscope-assisted ICH evacuation is a safe and effective treatment for selected patients with ICH. The rebleeding, morbidity, and mortality rates of our technique were lower than the reported outcomes of traditional craniotomy.

## Data Availability Statement

The original contributions presented in the study are included in the article/Supplementary Material, further inquiries can be directed to the corresponding author.

## Ethics Statement

The studies involving human participants were reviewed and approved by Institutional Review Board of National Taiwan University Hospital. Written informed consent for participation was not required for this study in accordance with the national legislation and the institutional requirements.

## Author Contributions

AH contributed to conceptualization, methodology, and formal analysis. C-HH and S-CC investigated the study. L-TK, S-JH, S-HY, and D-ML contributed to resources. C-HH wrote the original draft and writing, reviewing, and editing the manuscript. All authors have read and agreed to the published version of the manuscript.

## Conflict of Interest

The authors declare that the research was conducted in the absence of any commercial or financial relationships that could be construed as a potential conflict of interest.

## Publisher's Note

All claims expressed in this article are solely those of the authors and do not necessarily represent those of their affiliated organizations, or those of the publisher, the editors and the reviewers. Any product that may be evaluated in this article, or claim that may be made by its manufacturer, is not guaranteed or endorsed by the publisher.

## References

[1] Gross BA Jankowitz BT Friedlander RM. Cerebral intraparenchymal hemorrhage: a review. Jama. (2019) 321:1295itz B 10.1001/jama.2019.2413 30938800

[2] Rennert RC Signorelli JW Abraham P Pannell JS Khalessi AA. Minimally invasive treatment of intracerebral hemorrhage. Expert Rev Neurother. (2015) 15:919rot. 10.1586/14737175.2015.1059755 26200128

[3] Vespa P Hanley D Betz J Hoffer A Engh J Carter R. ICES (Intraoperative Stereotactic Computed Tomography-Guided Endoscopic Surgery) for brain hemorrhage: a multicenter randomized controlled trial. Stroke. (2016) 47:2749 D, 10.1161/STROKEAHA.116.013837 PMC6168060 27758940

[4] Hanley DF Thompson RE Rosenblum M Yenokyan G Lane K McBee N. Efficacy and safety of minimally invasive surgery with thrombolysis in intracerebral haemorrhage evacuation (MISTIE III): a randomised, controlled, open-label, blinded endpoint phase 3 trial. Lancet. (2019) 393:1021son 10.1016/S0140-6736(19)30195-3 PMC6894906 30739747

[5] Tang Y Yin F Fu D Gao X Lv Z Li X. Efficacy and safety of minimal invasive surgery treatment in hypertensive intracerebral hemorrhage: a systematic review and meta-analysis. BMC Neurol. (2018) 18:136. 10.1186/s12883-018-1138-9 30176811 PMC6120062

[6] Kuo LT Chen CM Li CH Tsai JC Chiu HC Liu LC. Early endoscope-assisted hematoma evacuation in patients with supratentorial intracerebral hemorrhage: case selection, surgical technique, and long-term results. Neurosurg Focus. (2011) 30:E9. 10.3171/2011.2.FOCUS10313 21456936

[7] Scaggiante J Zhang X Mocco J Kellner CP. Minimally invasive surgery for intracerebral hemorrhage. Stroke. (2018) 49:2612–20. 10.1161/STROKEAHA.118.020688 30355183

[8] Xia Z Wu X Li J Liu Z Chen F Zhang L. Minimally invasive surgery is superior to conventional craniotomy in patients with spontaneous supratentorial intracerebral hemorrhage: A systematic review and meta-analysis. World Neurosurg. (2018) 115:266–73. 10.1016/j.wneu.2018.04.181 29730105

[9] Hemphill JC 3rd Greenberg SM Anderson CS Becker K Bendok BR Cushman M. Guidelines for the management of spontaneous intracerebral hemorrhage: a guideline for healthcare professionals from the American Heart Association/American Stroke Association. Stroke. (2015) 46:2032essi 10.1161/STR.0000000000000069 26022637

[10] Gregson BA Broderick JP Auer LM Batjer H Chen XC Juvela S. Individual patient data subgroup meta-analysis of surgery for spontaneous supratentorial intracerebral hemorrhage. Stroke. (2012) 43:1496–504. 10.1161/STROKEAHA.111.640284 22511006 PMC3419479

[11] Nishihara T Teraoka A Morita A Ueki K Takai K Kirino T. A transparent sheath for endoscopic surgery and its application in surgical evacuation of spontaneous intracerebral hematomas. Technical note J Neurosurg. (2000) 92:1053–5. 10.3171/jns.2000.92.6.1053 10839271

[12] Chen CC Cho DY Chang CS Chen JT Lee WY Lee HC. A stainless steel sheath for endoscopic surgery and its application in surgical evacuation of putaminal haemorrhage. J Clin Neurosci. (2005) 12:937i. C 10.1016/j.jocn.2005.04.006 16275100

[13] Cho DY Chen CC Chang CS Lee WY Tso M. Endoscopic surgery for spontaneous basal ganglia hemorrhage: comparing endoscopic surgery, stereotactic aspiration, and craniotomy in noncomatose patients. Surg Neurol. (2006) 65:547–55. discussion 55–6. 10.1016/j.surneu.2005.09.032 16720167

[14] Hannah TC Kellner R Kellner CP. Minimally invasive intracerebral hemorrhage evacuation techniques: a review. Diagnostics. (2021) 11:576. 10.3390/diagnostics11030576 33806790 PMC8005063

[15] Gazzeri R Galarza M Neroni M Alfieri A Esposito S. Minimal craniotomy and matrix hemostatic sealant for the treatment of spontaneous supratentorial intracerebral hemorrhage. J Neurosurg. (2009) 110:939rza 10.3171/2008.8.JNS17642 19061356

[16] Nagasaka T Tsugeno M Ikeda H Okamoto T Takagawa Y Inao S. Balanced irrigation-suction technique with a multifunctional suction cannula and its application for intraoperative hemorrhage in endoscopic evacuation of intracerebral hematomas: technical note. Neurosurgery. (2009) 65:E826–7. discussion E7. 10.1227/01.NEU.0000350985.58062.3F 19834365

[17] Jimmerson C. Critical incident stress debriefing. J Emerg Nurs. (1988) 14:43A−5A. 3059021

[18] Kurtsoy A Oktem IS Koc RK Menku A Akdemir H Tucer B. Surgical treatment of thalamic hematomas via the contralateral transcallosal approach. Neurosurg Rev. (2001) 24:108em I 10.1007/PL00012391 11485230

[19] Kellner CP Chartrain AG Nistal DA Scaggiante J Hom D Ghatan S. The stereotactic intracerebral hemorrhage underwater blood aspiration (SCUBA) technique for minimally invasive endoscopic intracerebral hemorrhage evacuation. J Neurointerv Surg. (2018) 10:771. 10.1136/neurintsurg-2017-013719 29572265 PMC6278654

[20] Zhao J Zhou C. The protective and hemodynamic effects of dexmedetomidine on hypertensive cerebral hemorrhage patients in the perioperative period. Exp Ther Med. (2016) 12:2903 Th 10.3892/etm.2016.3711 PMC5103723 27882094

[21] Dong R Li F Xu Y Chen P Maegele M Yang H. Safety and efficacy of applying sufficient analgesia combined with a minimal sedation program as an early antihypertensive treatment for spontaneous intracerebral hemorrhage: a randomized controlled trial. Trials. (2018) 19:607. 10.1186/s13063-018-2943-6 30400977 PMC6219080

[22] Mayer SA Brun NC Begtrup K Broderick J Davis S Diringer MN. Efficacy and safety of recombinant activated factor VII for acute intracerebral hemorrhage. N Engl J Med. (2008) 358:2127C, B 10.1056/NEJMoa0707534 15728810

[23] Al Owesie RM Morton CS. Psychopharmacologic intervention after hemorrhagic basal ganglia damage. J Neurol Sci. (2012) 322:77Mor 10.1016/j.jns.2012.06.014 22795553

[24] Jeon DG Kim YW Kim NY Park JH. Serotonin syndrome following combined administration of dopaminergic and noradrenergic agents in a patient with akinetic mutism after frontal intracerebral hemorrhage: a case report. Clin Neuropharmacol. (2017) 40:180mac 10.1097/WNF.0000000000000220 28622210

[25] van Asch CJ Luitse MJ Rinkel GJ van der Tweel I Algra A Klijn CJ. Incidence, case fatality, and functional outcome of intracerebral haemorrhage over time, according to age, sex, and ethnic origin: a systematic review and meta-analysis. Lancet Neurol. (2010) 9:167.uit 10.1016/S1474-4422(09)70340-0 20056489

[26] Krishnan K Beishon L Berge E Christensen H Dineen RA Ozturk S. Relationship between race and outcome in Asian, Black, and Caucasian patients with spontaneous intracerebral hemorrhage: data from the virtual international stroke trials archive and efficacy of nitric oxide in stroke trial. Int J Stroke. (2018) 13:362–73. 10.1177/1747493017744463 29165060

[27] Gregson BA Mendelow AD. International variations in surgical practice for spontaneous intracerebral hemorrhage. Stroke. (2003) 34:2593del 10.1161/01.STR.0000097491.82104.F3 14563963

[28] Adeoye O Woo D Haverbusch M Sekar P Moomaw CJ Broderick J. Surgical management and case-fatality rates of intracerebral hemorrhage in 1988 and 2005. Neurosurgery. (2008) 63:1113–7. discussion 7–8. 10.1227/01.NEU.0000330414.56390.DE 19057323 PMC2717618

[29] Andaluz N Zuccarello M. Recent trends in the treatment of spontaneous intracerebral hemorrhage: analysis of a nationwide inpatient database. J Neurosurg. (2009) 110:403arel 10.3171/2008.5.17559 19249936

[30] Munakomi S Agrawal A. Advancements in managing intracerebral hemorrhage: transition from nihilism to optimism. Adv Exp Med Biol. (2019) 1153:1ol. 10.1007/5584_2019_351 30888664

[31] Hanley DF Thompson RE Muschelli J Rosenblum M McBee N Lane K. Safety and efficacy of minimally invasive surgery plus alteplase in intracerebral haemorrhage evacuation (MISTIE): a randomised, controlled, open-label, phase 2 trial. Lancet Neurol. (2016) 15:1228pson 10.1016/S1474-4422(16)30234-4 PMC5154627 27751554

[32] Prasad K Mendelow AD Gregson B. Surgery for primary supratentorial intracerebral haemorrhage. Cochrane Database Syst Rev. (2008) CD000200. 10.1002/14651858.CD000200.pub2 18843607

[33] Zhou X Chen J Li Q Ren G Yao G Liu M. Minimally invasive surgery for spontaneous supratentorial intracerebral hemorrhage: a meta-analysis of randomized controlled trials. Stroke. (2012) 43:2923 Li 10.1161/STROKEAHA.112.667535 22989500

[34] Jadhav V Zhang JH. Surgical brain injury: prevention is better than cure. Front Biosci. (2008) 13:3793 JH 10.2741/2968 18508474

[35] Morgenstern LB Demchuk AM Kim DH Frankowski RF Grotta JC. Rebleeding leads to poor outcome in ultra-early craniotomy for intracerebral hemorrhage. Neurology. (2001) 56:1294 De 10.1212/WNL.56.10.1294 11376176

[36] Nishihara T Nagata K Tanaka S Suzuki Y Izumi M Mochizuki Y. Newly developed endoscopic instruments for the removal of intracerebral hematoma. Neurocrit Care. (2005) 2:67re.g 10.1385/NCC:2:1:067 16174973

[37] Little AS Liu S Beeman S Sankar T Preul MC Hu LS. Brain retraction and thickness of cerebral neocortex: an automated technique for detecting retraction-induced anatomic changes using magnetic resonance imaging. Neurosurgery. (2010) 67:ons277–82. discussion ons82. 10.1227/01.NEU.0000374699.12150.0 20679923

[38] Miki K Yagi K Nonaka M Iwaasa M Abe H Morishita T. Spot sign as a predictor of rebleeding after endoscopic surgery for intracerebral hemorrhage. J Neurosurg. (2019) 130:1485–90. 10.3171/2017.12.JNS172335 29799345

[39] Li Q Zhang G Xiong X Wang XC Yang WS Li KW. Black hole sign: novel imaging marker that predicts hematoma growth in patients with intracerebral hemorrhage. Stroke. (2016) 47:1777Xion 10.1161/STROKEAHA.116.013186 27174523

[40] Kerboul B Courtois B. [Segmental posterior spinal osteosynthesis using the Luque-Dove technic]. J Chir. (1989) 126:193toi 2732280

[41] Kim JE Ko SB Kang HS Seo DH Park SQ Sheen SH. Clinical practice guidelines for the medical and surgical management of primary intracerebral hemorrhage in Korea. J Korean Neurosurg Soc. (2014) 56:175surg 10.3340/jkns.2014.56.5.452 PMC4217052 25368758

