# Supplementary material for: Minimally Invasive Neurosurgery for Spontaneous Intracerebral Hemorrhage—10 Years of Working Progress at National Taiwan University Hospital
Source: Front Neurol. 2022 May 20;13:817386. doi: 10.3389/fneur.2022.817386 (PMC9163304; doi:10.3389/fneur.2022.817386)
Supplement: Supplementary file 1 [file Table_1.DOCX]

Supplementary Material

# Supplementary Tables

Supplementary table 1. Summary early endoscopic cases with extremely low rebleeding rate

| Authors & Year | Indication | Timing | Pt characteristics | Technique | Evacuation rate | Rebleeding rate | Long-term outcome |
| --- | --- | --- | --- | --- | --- | --- | --- |
| Nishihara et al., 2005 (24) | Putaminal, thalamic, & subcortical ICH w/ vol >20 & cerebellar ICH w/ vol >15 mL w/ deterioration of consciousness | ultra-early op (w/in 3 h) for hemorrhages w/ vol >30 mL or hemorrhages causing impending herniation | 82 pts w/ ICH or IVH; 44 w/ putaminal ICH, 12 w/ thalamic ICH, 8 w/ subcortical ICH, 8 w/ cerebellar ICH, 10 w/ IVH | transparent sheath; hemostasis by electric coagulation at suction end; transparent cap attached to flexible endoscope provides clear visualization of op field during hematoma evacuation, which can prevent injury of ventricular walls | 96% (range 86%– 100%) | no postop re-bleeding | NA |
| Chen et al., 2005 (25) | Putaminal ICH vol >20 ml, GCS 5–12 w/ focal neurol deficit | 1–5 h (median 2 h) | 7 pts w/ hypertensive putaminal ICH; age range: 45–69 yrs | an 11-cm-long SS tube was adapted to serve as endoscopic sheath; op route along long axis of hematoma, requiring frontal approach | 90%–97% (median 93%); ICH vol 20– 180 ml (median 78 ml) preop, 2–16 mL (median 6 ml) postop | no postop re-bleeding | 6 pts were fully independent, including 4 who had no residual disability & 2 who had mod disability; 1 pt remained in a persistent vegetative state at clinical FU after 6 months |
| Nagasaka et al., 2009 (11) | Putaminal ICH vol >31 ml, cerebellar ICH w/ diam >3 cm, or thalamic ICH w/ vol >20 ml & acute hydrocephalus | median time to op: 4 h | 23 pts; 15 w/ putaminal ICH, 6 w/ cerebellar ICH, 2 w/ thalamic ICH; mean age 61.4 yrs (range 36–85 yrs); mean preop GCS score: 7.2 (range 4–13) | a combination irrigation-coagulation suction cannula or multifunctional suction cannula was used | 99% | 0% | long-term outcome not mentioned, but the rate of good recovery & mod disability) at discharge was 17.3% |
| Kuo et al., 2011 (6) | Putaminal ICH vol >30 ml, or thalamic ICH vol >20 ml & IVH w/ acute hydrocephalus, or subcortical ICH vol >30 ml w/ sig mass effect & neurol deterioration | all w/in 12 h & 84% performed w/in 4 h | 68 pts; 35 w/ putaminal ICH, 24 w/ thalamic ICH, & 9 w/ subcortical ICH; mean age 63 yrs (range 42–82 yrs); mean preop GCS score 7.1 (range 4–14) | transparent sheath, balanced irrigation-suction technique for identification of bleeder, coagulation w/ suction bipolar coagulator, hemostasis w/ Floseal; endoscope was an option | 93%; putaminal ICH 96%; thalamic ICH 86%; subcortical ICH 98% | 1.5% | mean GCS score was 11.6 & GOSE was 4.9 at 6-mo FU |
| Luh et al., 2018 (9) | Putaminal ICH vol >30 ml, or thalamic ICH vol >20 ml & IVH w/ acute hydrocephalus, or subcortical ICH vol >30 ml w/ sig mass effect & neurol deterioration | all w/in 12 h | 42 pts; 23 w/ putaminal ICH, 16 w/ thalamic ICH, 3 w/ subcortical ICH; mean age 59 yrs (range 37–74 yrs); mean preop GCS score 7.86 (range 4–14) | transparent sheath, balanced irrigation-suction technique for identification of bleeder, coagulation w/ suction bipolar coagulator, hemostasis w/ Floseal | 80.8%; putaminal ICH 92.2%; thalamic ICH 63.3%; subcortical ICH 86.6% | 4.8% | mean GCS score was 12.77 & GOSE was 4.55 at 6-mo FU |
| Miki et al., 2018 (36) | ICH w/ sig mass effect & neurol deterioration; putaminal ICH > 10 mL w/ IVH and hydrocephalus; cerebellar ICH diameter > 3 cm | 127 pts; 64 < 4 h, 29 between 4 – 8 h, 34 > 8 h or unknown time | 127 pts; 93 w/ basal ganglia ICH, 14 w/ subcortical ICH, 20 w/ cerebellar ICH; mean age 66.7 yrs; mean preop GCS score 9 | burr hole or small craniotomy, transcortical or transventricular approach | 83.4% pts achieve successful removal (> 80% hematoma) | 8.7%; 20.8% (5 of 24) w/ spot sign, 5.8% (6 of 103) w/o spot sign | NA |
| Present series | Putaminal ICH vol >30 ml, or thalamic ICH vol >20 ml & IVH w/ acute hydrocephalus, or subcortical ICH vol >30 ml w/ sig mass effect & neurol deterioration | all w/in 8 h | 76 pts; 56 w/ putaminal ICH, 9 w/ thalamic ICH, 11 w/ subcortical ICH; mean age 59.2 yrs; mean preop GCS score 9.99 (range 4–14) | transparent sheath, balanced irrigation-suction technique for identification of bleeder, coagulation w/ suction bipolar coagulator, hemostasis w/ Floseal | 79.4%; putaminal ICH 81.6%; thalamic ICH 70.55%; subcortical ICH 75.44% | 3.9% | mean GCS score was 11.77 & GOSE was 3.37 at 6-mo FU |
